# Relationship between mandibular arch shape and skeletal jaw base in various growth patterns among north Maharashtrian population

**DOI:** 10.6026/973206300213692

**Published:** 2025-10-31

**Authors:** Hrushikesh Aphale, Payal Barse, Sunilkumar Nagmode, Kunal Patil, Vivek Shinde, Pranoti Gade

**Affiliations:** 1 Department of Orthodontics and Dentofacial Orthopaedics, SMBT Dental college and Hospital Ghulewadi, Sangamner, Maharashtra, India

**Keywords:** Arch form, skeletal jaw base, orthodontic treatment, growth patterns

## Abstract

Achieving stable orthodontic results requires an understanding of the relationship between the skeletal jaw base and the shape of the mandibular arch across different growth patterns. Therefore, it is of interest to analyse 63 North Maharashtrian subjects grouped by horizontal, average and vertical growth patterns and skeletal classifications I, II and III using cephalometric parameters (SN-MP, ANB and Wits index). Cephalometric and dental cast measurements were recorded and statistically analyzed using SPSS version 24. Statistically significant associations were found between growth patterns and several skeletal variables such as OP-MP angle and Jaraback ratio but, skeletal classifications showed no consistent correlation with mandibular arch width. Thus, we show that while certain skeletal parameters vary with growth patterns, mandibular arch shape does not reliably correlate with skeletal jaw base classification, highlighting the need for individualized orthodontic assessment.

## Background:

The human dental arch develops through a complex interaction between skeletal growth and neuromuscular activity, influenced by both genetic and epigenetic factors [1]. The mandible grows by remodeling the glenoid fossa and altering the dynamics of the muscles used in mastication [2]. The growth pattern-horizontal, average, or vertical-is determined in part by the mandibular plane orientation (Go-Gn) in relation to the cranial base (SN) [3]. Previous studies suggest that facial divergence influences transverse jaw dimensions. For instance, dolichofacial individuals with increased SN-MP angles tend to have narrower transverse widths [4]. However, conflicting studies like Haje *et al.* have not found a substantial correlation between transverse relationships and mandibular arch form [5]. Although class II and III malocclusions have been analyzed in terms of vertical and sagittal growth patterns, the specific relationship between mandibular arch shape and skeletal base remains underexplored. The integration of cephalometric analysis with arch form measurements offers insights into skeletal-dental harmony. Therefore, it is of interest to evaluate these relationships systematically within a defined ethnic group-North Maharashtrian individuals-to potentially guide more culturally specific orthodontic treatment plans. Customizing treatment plans for improved function and stability following orthodontics may also be made simpler with an understanding of this connection.

## Methodology:

This retrospective study was approved by the Institutional ethics and Review Board [ethical number] and was conducted in Orthodontic and Dentofacial Orthopedics department. Sample size estimation was based on the previous studies [6], resulting in 63 participants (N=63), grouped into 9 subgroups based on skeletal class (I, II, III) and growth pattern (horizontal, average, vertical), with 7 samples per group.

## Inclusion and exclusion criteria:

Inclusion criteria involved patients with fully erupted permanent incisors and first molars and SN-MP angle between 25° and 38°. Patients having standardized lateral cephalograms with no TMJ disorders or previous orthodontic treatment were also included. Exclusion criteria included patients having crossbite or scissor bite, craniofacial anomalies, previous trauma and molar rotation or destructive caries.

## Data collection and statistical analysis:

Each subject's data included lateral cephalogram, orthopantomogram, intraoral/extraoral photographs and study models. Cephalometric analyses measured skeletal parameters (SN-MP, OP-MP, PP-OP, ANB, Wits index, etc.). The landmarks and reference lines are given in Figure 1 (see PDF) and described in Table 1 (see PDF). Acetate tracing, scale, divider and cephalostat were used on standardized radiographs. Dental measurements which included anterior and posterior mandibular widths from dental casts are demonstrated in Figure 2 (see PDF). To minimize error, one trained operator measured each parameter twice. Statistical analysis was performed using SPSS (v 24.0, IBM). Intergroup comparisons were conducted with t test and ANOVA was used for intragroup comparison. P-values <0.05 were considered significant.

## Results:

A total of 63 subjects were divided into 9 subgroups based on growth pattern (horizontal, average, vertical) and skeletal class (I, II, III), with 7 subjects per group. Cephalometric and mandibular arch width measurements were compared both between skeletal classes within each growth pattern (intergroup analysis) and across growth patterns within each skeletal class (intragroup analysis). In the horizontal growth pattern (Table 2 - see PDF), statistically significant differences were observed for OP-MP (p=0.002), Wits Index (p<0.001) and ANB angle (p<0.001), indicating skeletal discrepancy particularly between Class II and Class III subjects. The remaining parameters, including SN-MP, PP-MP, PP-OP, Lower Gonial Angle and Jarabak Ratio, showed no statistically significant differences (p>0.05). In the average growth pattern (Table 3 - see PDF), significant interclass variation was again noted in Wits Index (p<0.001) and ANB (p<0.001), while Lower Gonial Angle showed marginal significance (p=0.043). Other parameters did not differ significantly. Among subjects with a vertical growth pattern (Table 4 - see PDF), statistically significant differences were found in OP-MP (p<0.001), Jarabak Ratio (p=0.022), Wits Index (p<0.001) and ANB (p<0.001). These findings highlight that the vertical grower's exhibit pronounced anteroposterior discrepancies, particularly among skeletal Class II and III patients. Transverse dimensions including anterior mandibular width, posterior mandibular width and posterior width at the distobuccal cusp showed no significant interclass differences in any growth pattern group (p>0.05), though a trend toward reduced posterior width in Class II was noted. In class I malocclusion, the pie chart (Figure 3 - see PDF), showed significant differences in SN-MP (p=0.018), PP-MP (p=0.004) and OP-MP (p=0.012), indicating changes in mandibular plane inclination across growth patterns. Other parameters such as ANB, Wits Index and transverse widths showed no significant variation. The pie chart (Figure 3 - see PDF) in Class II malocclusion, significant differences were seen in PP-OP (p=0.003), Lower Gonial Angle (p=0.011) and Jarabak Ratio (p<0.001), suggesting a steeper palatal plane and altered mandibular morphology in vertical growers. The pie chart (Figure 3 - see PDF) for Class III malocclusion, significant differences were observed in OP-MP (p=0.021), PP-OP (p=0.004), Lower Gonial Angle (p<0.001), Wits Index (p<0.001) and ANB (p<0.001), reflecting the influence of vertical growth pattern on both vertical and anteroposterior skeletal relationships. Among transverse measurements, anterior mandibular width varied significantly across growth patterns in Class II (p=0.011), whereas other transverse parameters showed no statistically significant intragroup differences (p>0.05).

## Discussion:

The present research provides insights into the complex relationship between skeletal growth patterns and mandibular arch form. While certain cephalometric variables like OP-MP angle and ANB showed statistically significant differences across growth types, mandibular arch widths (anterior and posterior) did not consistently correlate with skeletal class or growth pattern. These findings are congruent with those of Haje *et al.* [5] and Saffarshahroudi *et al.* [7], who reported no direct association between transverse arch shape and sagittal relationships. In horizontal growth patterns, OP-MP showed significant variation (p=0.002), indicating potential differences in vertical occlusal dynamics. In average and vertical patterns, the Jaraback ratio and lower gonial angle showed differences between classes, reflecting skeletal compensations. However, despite these skeletal differences, arch width remained statistically nonsignificant across classes and growth patterns. This suggests that Local factors like habits and musculature may have a greater influence on the shape of the dental arch than by skeletal base alone. Similar conclusions were made by Singh *et al.* [8] and Domenico *et al.* [6] in their studies. Intragroup comparisons within Class I, II and III malocclusion also revealed variations in cephalometric angles but no definitive pattern in mandibular arch width. For instance, the mandibular arch widths of Class II vertical pattern subjects did not differ significantly, but they did have higher OP-MP angles and lower Jaraback ratios, which indicate increased vertical facial growth. Kadam *et al.* [9] similarly reported that vertical and horizontal grower's exhibit significant differences in mandibular dimensions and airway space, yet arch width did not necessarily parallel these skeletal variations. This dissociation between skeletal base and arch width draws attention to an important orthodontic consideration. While skeletal diagnosis is crucial for treatment planning, arch form customization should not rely solely on skeletal classification. In orthodontic therapy, arch form templates may need to be customized for each patient, especially in populations with significant ethnic variation [8]. The differences observed in SN-MP and PP-MP among the three growth patterns reaffirms previous literature that vertical growth patterns show greater divergence [10]. Yet, mandibular width measurements did not parallel these skeletal differences. Such outcomes reinforce the idea that skeletal and dental features, though interrelated, are not universally correlated. Edward Harris and Michelle Johnson's work aligns with these observations, suggesting that skeletal (craniometric) variables have higher heritability, whereas dental positional traits are more environment-driven [11]. Moreover, the significant changes in OP-MP and ANB angles among various groups, particularly in Class III subjects, underline the rotational effects of mandibular growth, which may affect occlusion but not necessarily alter the arch width. Vertical growers, often presenting with a more open mandibular morphology, might still retain average arch dimensions [12]. The lack of consistent correlation challenges assumptions in standard orthodontic treatment planning and emphasizes a multi-dimensional, individualized approach. Future studies with larger, multicentric samples and three-dimensional imaging might help further clarify the skeletal-dental interrelationship.

## Conclusion:

Mandibular arch width measurements did not demonstrate a consistent correlation with skeletal jaw base or growth pattern within the North Maharashtrian population. Cephalometric parameters showed significant variation across growth patterns and skeletal classifications. Orthodontic treatment planning should incorporate both skeletal and individual dental arch considerations rather than rely solely on growth patterns or cephalometric norms for optimal orthodontic outcomes.

## References

[1] Harris EF, Johnson M.G (1991). Am J Orthod Dentofacial Orthop..

[2] Enlow DH, Moyers RE (1971). J Am Dent Assoc..

[3] Benedicto EN (2016). J Dent..

[4] Nasby JA (1972). Angle Orthod..

[5] El Haje OA (2014). J Contemp Dent Pract..

[6] Ciavarella D (2023). Medicina (Kaunas)..

[7] Shahroudi AS, Etezadi T. (2013). Journal of dentistry (Tehran, Iran)..

[8] Singh A (2023). World J Dent..

[9] Kadam V (2021). IP Indian J Orthod Dentofacial Res..

[10] Knigge Ryan P (2021). Am J Orthod Dentofacial Orthop..

[11] Harris EF, Johnson. M.G (1991). Am J Orthod Dentofacial Orthop..

[12] Grippaudo C (2013). Prog Orthod..

